# Examining Social Cognition with Embodied Robots: Does Prior Experience with a Robot Impact Feedback-associated Learning in a Gambling Task?

**DOI:** 10.5334/joc.167

**Published:** 2021-05-31

**Authors:** Abdulaziz Abubshait, Craig G. McDonald, Eva Wiese

**Affiliations:** 1 George Mason University, US; 2 Italian Institute of Technology, IT; 3 Berlin Institute of Technology, US

**Keywords:** Cognitive Control, Learning, Reward processing, Social cognition

## Abstract

Social agents rely on the ability to use feedback to learn and modify their behavior. The extent to which this happens in social contexts depends on motivational, cognitive and/or affective parameters. For instance, feedback-associated learning occurs at different rates when the outcome of an action (e.g., winning or losing in a gambling task) affects oneself (“Self”) versus another human (“Other”). Here, we examine whether similar context effects on feedback-associated learning can also be observed when the “other” is a social robot (here: Cozmo). We additionally examine whether a “hybrid” version of the gambling paradigm, where participants are free to engage in a dynamic interaction with a robot, then move to a controlled screen-based experiment can be used to examine social cognition in human-robot interaction. This hybrid method is an alternative to current designs where researchers examine the effect of the interaction on social cognition during the interaction with the robot. For that purpose, three groups of participants (n total = 60) interacted with Cozmo over different time periods (no interaction vs. a single 20 minute interaction in the lab vs. daily 20 minute interactions over five consecutive days at home) before performing the gambling task in the lab. The results indicate that prior interactions impact the degree to which participants benefit from feedback during the gambling task, with overall worse learning immediately after short-term interactions with the robot and better learning in the “Self” versus “Other” condition after repeated interactions with the robot. These results indicate that “hybrid” paradigms are a suitable option to investigate social cognition in human-robot interaction when a fully dynamic implementation (i.e., interaction and measurement dynamic) is not feasible.

## Introduction

As social creatures, a considerable part of our lives revolves around interactions with other human beings. However, due to an increased availability of artificially intelligent agents in modern societies, our future social interactions will likely expand to nonhuman agents like avatars or robots (Wiese et al., 2017). In fact, robots have already been implemented in elderly care as social assistants to increase emotional comfort (e.g,. Birks et al., 2016; Tapus et al., 2007), in therapeutic settings with children with autism spectrum disorder to train social-cognitive skills (Bekele et al., 2014; Warren et al., 2015), as well as in rehabilitation settings to improve sensorimotor skills (Basteris et al., 2014). Nonetheless, despite considerable progress in equipping artificial agents with social capabilities, they are still limited in their ability to interact with humans in a natural way (Wiese et al., 2017), and the public remains skeptical concerning the introduction of robot assistants to everyday life (Bartneck & Reichenbach, 2005). Specifically, the use of robots as companions for children has been discussed controversially due to concerns about privacy, fear of reduced interest in human-human interactions due to increased attachment to the robot companion, and anticipation of negative impacts on learning outcomes and development (Sharkey, 2016). While these concerns should be taken seriously, there is a lack of empirical studies that systematically examine the impact of robots on social cognition, development and wellbeing; this is particularly true with regard to long-term social interactions in everyday environments. Since robots will employ social roles in our society and share our environments with us in the future, it is essential to understand how to design them so that they can foster rewarding long-term social interactions by activating relevant social schemes, behaviors and emotions without negatively impacting human-human interactions.

To examine whether and how humans develop social attachments to robot companions, experimental paradigms need to (i) adequately reflect the dynamics of long-term social interactions with embodied robots in everyday environments (i.e., external validity) but at the same time (ii) allow for controlled and reproducible measures of social cognition (i.e., internal validity). Most importantly, behavioral and neurophysiological studies have shown that social-cognitive mechanisms unfold differently in dynamic or “online” interactions (in contrast to the simulation of social interactions on the screen or “offline” paradigms), and activate brain areas involved in social cognition differently (Schilbach et al., 2013). For instance, while offline paradigms have shown that mechanisms of social attention (i.e., the extent to which observers shift their attention to locations that are gazed-at (see Frischen et al., 2007; for a review) differ between human-human and human-robot interaction (Admoni et al., 2011), online paradigms examining the same mechanisms in face-to-face interactions using embodied robot platforms show that these mechanisms resemble human-human interaction more closely than originally thought (Kompatsiari et al., 2018). It was also shown that depending on whether “offline” or “online” paradigms are employed, different brain regions are implicated in social attention: offline fMRI studies identify brain regions in the right hemifield as important neural correlates of social attention (Capozzi & Ristic, 2020; for a review), whereas online studies that use face-to-face paradigms implicate structures in the left hemifield (e.g., Cole et al., 2016). These studies show that in order to get a realistic idea of how social-cognitive mechanisms are engaged in human-robot interaction, paradigms should include an “online” component, such that at least one part of the experiment should allow participants to engage in dynamic interactions with an embodied robot.

Longer-term social interactions with others require monitoring the behavior of others and adjusting our behaviors to theirs to ensure successful exchanges of knowledge, affiliation and support (Insel & Fernald, 2004). In order to be able to adapt behaviors to ever-changing environments and learn from previous experiences, we rely on feedback of others to tell the difference between behavioral responses that are appropriate and those that are not. Receiving positive feedback (e.g., smile) positively reinforces a given behavior and increases the likelihood that it will be shown again in the future; negative feedback (e.g., frown) negatively reinforces a given behavior and decreases the likelihood that it will be shown again in the future (Krigolson et al., 2009). There is also a direct link between feedback processing and learning, such that the response to feedback is stronger (or weaker) the less (or more) advanced the learning progress is: participants use feedback to map their expectations regarding an outcome to the actual outcome, with positive feedback indicating a good match (and positively reinforcing an expectation) and negative feedback indicating a bad match (and negatively reinforcing an expectation) between expectation and outcome (i.e., reinforcement learning; Holroyd & Coles, 2002; Schultz, 2017). While learners rely on feedback at the beginning of the learning process, they depend increasingly less on feedback in the later stages of a learning process but place more weight on their own responses to inform upcoming behaviors (Holroyd & Coles, 2002; Krigolson et al., 2009). This suggests that how we learn in social interactions is a consequence of how we process (i.e., feedback monitoring) and are reinforced by others’ feedback (i.e., reinforcement learning; Hobson & Inzlicht, 2016). While feedback processing is relatively well understood in human-human interactions, it has not been examined in human-robot interactions yet (to our knowledge). This is of particular importance, however, given that robots are already used in educational and therapeutic settings (and will be used even more in these fields in the future; Rahwan et al., 2019) where a reduced response to feedback could have measurable negative effects on learning outcomes. Feedback monitoring and reward processing are also linked to prosocial behavior (de Bruijn et al., 2017), and as such utterly important for supporting positive long-term human-robot interactions.

One paradigm that has traditionally been used to examine feedback processing (Gehring & Willouby, 2002) and has been adapted to investigate social reinforcement learning is the gambling task (e.g., Hassall et al., 2016; Kimura & Katayama, 2016; Krigolson et al., 2013; Lemoine & Roland-Lévy, 2017; Leng & Zhou, 2010; Rigoni et al., 2010). In one variant of this task, participants are asked to gamble by picking (on a trial-by-trial basis) one of two differently colored squares (one of which is associated with a higher chance of winning than the other one) shown next to each other on a screen over a consecutive sequence of trials. After each selection, participants receive feedback whether they won (“Win”, i.e., positive feedback) or lost (“Lose”, i.e., negative feedback) this trial. Electrophysiological studies using this task have shown that event-related potentials (ERPs) implicated in reward processing, such as the *Feedback-Related Negativity* (FRN) and/or *Reward Positivity* (RewP), are more pronounced when gambling for oneself (“Self”) versus another person (“Other”; Hassall et al., 2016; Krigolson et al., 2013). The extent to which participants learn to select the option that is associated with a higher chance of winning can be predicted by the extent to which participants process feedback, such that a stronger reliance on feedback cues is associated with worse learning outcomes (Lohse et al., 2020).

Importantly, these mechanisms are sensitive to the context in which feedback is provided. For instance, feedback processing is altered by whether a person’s performance has a direct impact on a partner’s performance (de Bruijn et al., 2011; Koban et al., 2012; Koban et al., 2013), whether the interaction is cooperative or competitive (Czeszumski et al., 2019; de Bruijn et al., 2011; Radke et al., 2011; Van Meel & Van Heijningen, 2010) or whether a social interaction partner is present during the delivery of feedback (Simon et al., 2014). Most importantly, the relationship between the gambler and the recipient of the gambling outcome (i.e., the “Other” in the gambling task) modulates feedback processing, such that when strangers are the recipients of winning outcomes, feedback processing is attenuated compared to when participants themselves receive the outcome (e.g., Hassall, Silver, Turk, & Krigolson, 2016; Krigolson, Hassall, Balcom & Turk, 2013). The presence of a social ingroup member (as opposed to a social outgroup member) also attenuates Self-Other differences, as the motivation to win is higher for a social agent that is believed to be similar to oneself (versus dissimilar; Hobson & Inzlicht, 2016). In line with this notion, there is no observable Self-Other difference in feedback processing when the recipient of a positive outcome is a friend versus a stranger (Leng & Zhou, 2010) indicating that feedback processing is sensitive to the social relationship between the gambler and the recipient of the outcome.

These findings suggest that feedback processing may differ in human-human- vs. human-robot interaction, partially because nonhuman social entities are often perceived as outgroup members (Hackel et al., 2014); they also suggest that similar to human-human interactions, feedback processing in human-robot interaction may change over time if participants start to get attached to robots and perceive them as friends. Support for this assumption comes from a recent neurophysiological study using the gambling task to examine whether familiarization with the robot Cozmo^1^ modulates feedback processing after a one-time interaction: one group of participants engaged in a 20-minutes interaction with Cozmo (playing interactive games with the robot via the associated mobile app) and the other group played the Simon Says game (an interactive game that does not involve Cozmo) before gambling for themselves (“Self”) or Cozmo (“Other”). The data shows larger RewP amplitudes and slower learning of the contingencies between color and chance of winning after one-time familiarization with Cozmo (Abubshait et al., 2021). Although this effect was not specific to the “Other” condition, but affected the “Self” condition as well, the results indicate that familiarization with a social robot does indeed modulate feedback-based learning in short-term interactions. What causes this effect and whether similar observations can be made for longer interactions durations warrants further investigation.

The goal of this paper is to present a proof-of-concept that hybrid paradigms that separate a dynamic, relatively unscripted interaction component (here: interaction with Cozmo via its associated app) from a highly controlled, scripted data collection component are suitable to investigate mechanisms of social cognition in human-robot interaction. Specifically, we explore how different degrees of familiarity with a robot due to prior interactions of different durations impact behavioral correlates of reward processing in form of feedback-associated learning in a gambling task (see Abubshait et al., 2021). For that purpose, we compare feedback-associated learning (i.e., speed with which participants learn to pick the square associated with the higher chance of winning) after a ***one-time interaction*** in the lab (about 20 minutes) to feedback-associated learning after ***repeated interactions*** with Cozmo in participants’ homes (over five consecutive days); both conditions are compared to a ***no interaction*** condition, in which participants do not familiarize themselves with Cozmo before performing the gambling task but instead play an interactive game that does not involve Cozmo (i.e., *Simon says*). In all three conditions, the participants then perform a computer-based version of the gambling task with Cozmo sitting underneath the screen in the participants’ field of view, making occasional eye blinks to signal that it is “on”.

This setup creates high external validity by allowing unrestricted social interactions with Cozmo to occur AND high internal validity due to the controlled setting in which reward processing is examined. As feedback-associated learning is impacted by a range of factors including social (e.g., social closeness between gambler and recipient; Leng & Zhou, 2010), motivational (e.g., better learning when intrinsically motivated; Wilhelm et al., 2019) and physiological (e.g., arousal: worse learning in engaging vs. calm environments; Lohse et al., 2020; Wilhelm et al., 2019) factors, it is challenging to formulate specific directed hypotheses regarding the effect of familiarization duration on reward processing. We generally expect that increased intrinsic motivation to perform well for Cozmo due to increased familiarity with the robot after longer initial familiarization, should positively affect feedback processing and lead to more effective learning of the association between “color” and “chance of winning” during gambling. Increased levels of arousal, in contrast, for instance due to an increased level of engagement, should negatively impact feedback processing and learning.

## Methods & Materials

### Participants

60 participants were recruited from George Mason University’s undergraduate participant pool (Mean Age = 22.34, Range = 18–55, SD = 5.9, 38 females) in exchange for course credit or monetary compensation. Participants have neither interacted with nor owned a Cozmo before participating in the study. Participants were assigned to the no-interaction condition (NI; n = 16), the one-time interaction condition (OTI; n = 17) or the repeated-interaction condition (RI; n = 18). All subjects were right-handed, had normal or corrected-to-normal vision.^2^ The data of several participants were excluded from the final analyses due to technical difficulties (n = 3) or participants not following the task protocol (n = 2) or not completing the study (n = 2); two additional participant data sets were excluded due to corrupted data files. Data handling and collection was in accordance to George Mason University’s ethics board. Raw data can be found on the OSF page of the experiment: *osf.io/6dfky/*.

### Apparatus

**Figure 1:**
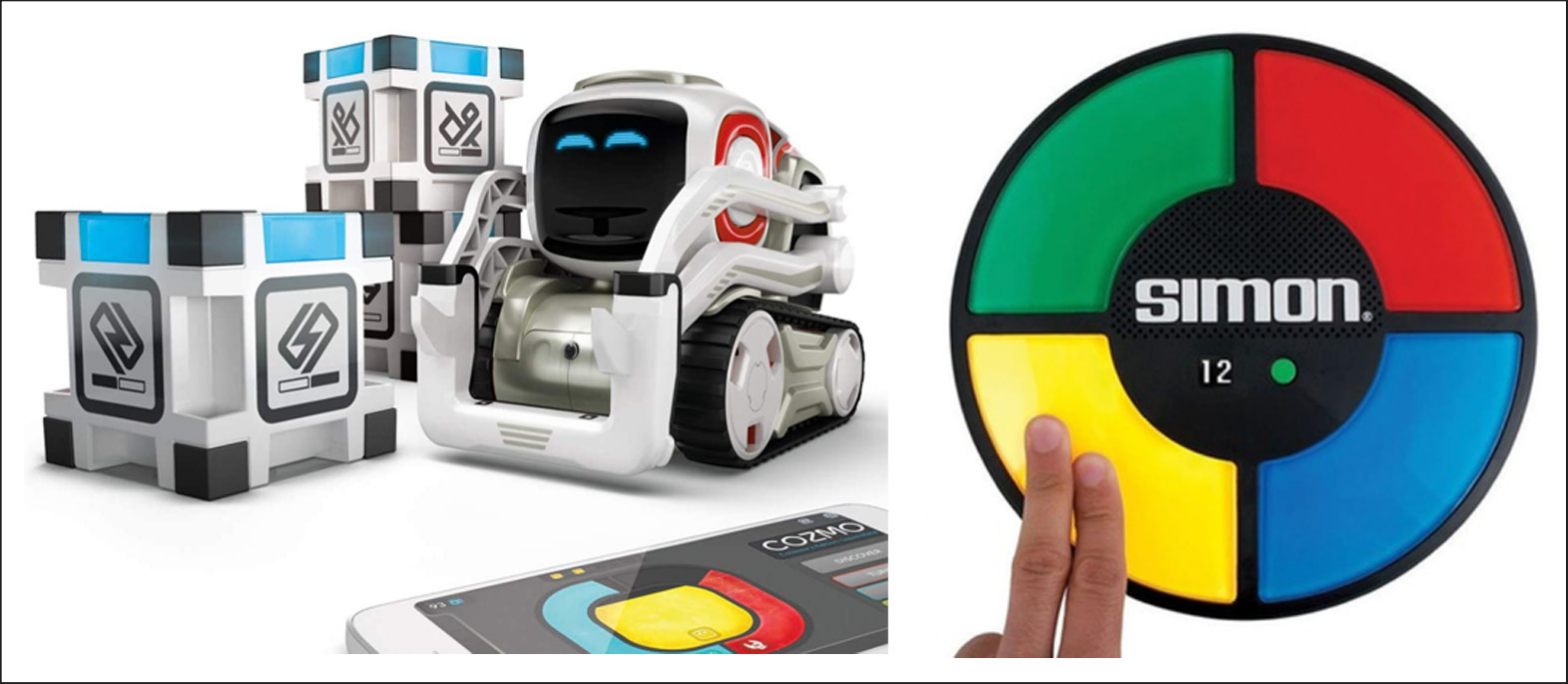
**The Cozmo robot and the Simon Says game:** Cozmo (used for one-time and repeated interaction conditions) is a tank-like robot with a screen-like face and blue squares as eyes. It can express a wide range of emotions using its eyes and – in addition – sounds. More information on the robot Cozmo can be found here: www.digitaldreamlabs.com/pages/cozmo. The Simon Says game (used for the no social interaction condition) is an electronic game that is equipped with pressable buttons that light up. When playing Simon Says, players see a series of buttons light up that are associated with a tone in a specific sequence that the player needs to mimic. After the player successfully mimics the sequence, the sequence would increase in length. The Simon Says game was chosen due to its resemblance to the Quick-tap game.

In the social interaction conditions (OTI, RI), participants interacted with the social robot Cozmo (Digital Dreamlabs, USA, version 3.2.0) using a Samsung Galaxy tablet (***Figure 1***). Cozmo is equipped with three cubes that are used to play interactive games with the robot (e.g., tapping a cube as fast as possible); all interactions are pre-programmed using the Cozmo app. In the no-interaction condition (NI), participants played the Simon Says game (Hasbro, Inc, USA), an engaging task that requires them to respond to different color patterns (***Figure 1***). The gambling task was programmed and presented using Matlab (Mathworks, Natick, MA) and Psychtoolbox (Brainard, 1997). Analyses were conducted using R (version 3.6), including the packages tidyverse (Wickham et al., 2019) and lme4 (Bates et al., 2014).

### Procedure

After providing consent, all participants were given a cover story that the experiment was a collaborative effort between the psychology and engineering departments at GMU. Specifically, they were told that “the engineering department was trying to decide which of their robots to upgrade and that the psychology department is helping them make that decision by conducting user-centered testing”. Afterwards, participants were introduced to Cozmo and familiarized with its basic functionalities. All participants were instructed that the latter part of the study will include a part where they will play a gambling task where they would be gambling for a chance to win a gift card for themselves (“Self”) for half of the blocks, and gambling for new hardware and software upgrades for Cozmo (e.g., batteries, “Cozmo”) for the other half of the blocks.

**Figure 2:**
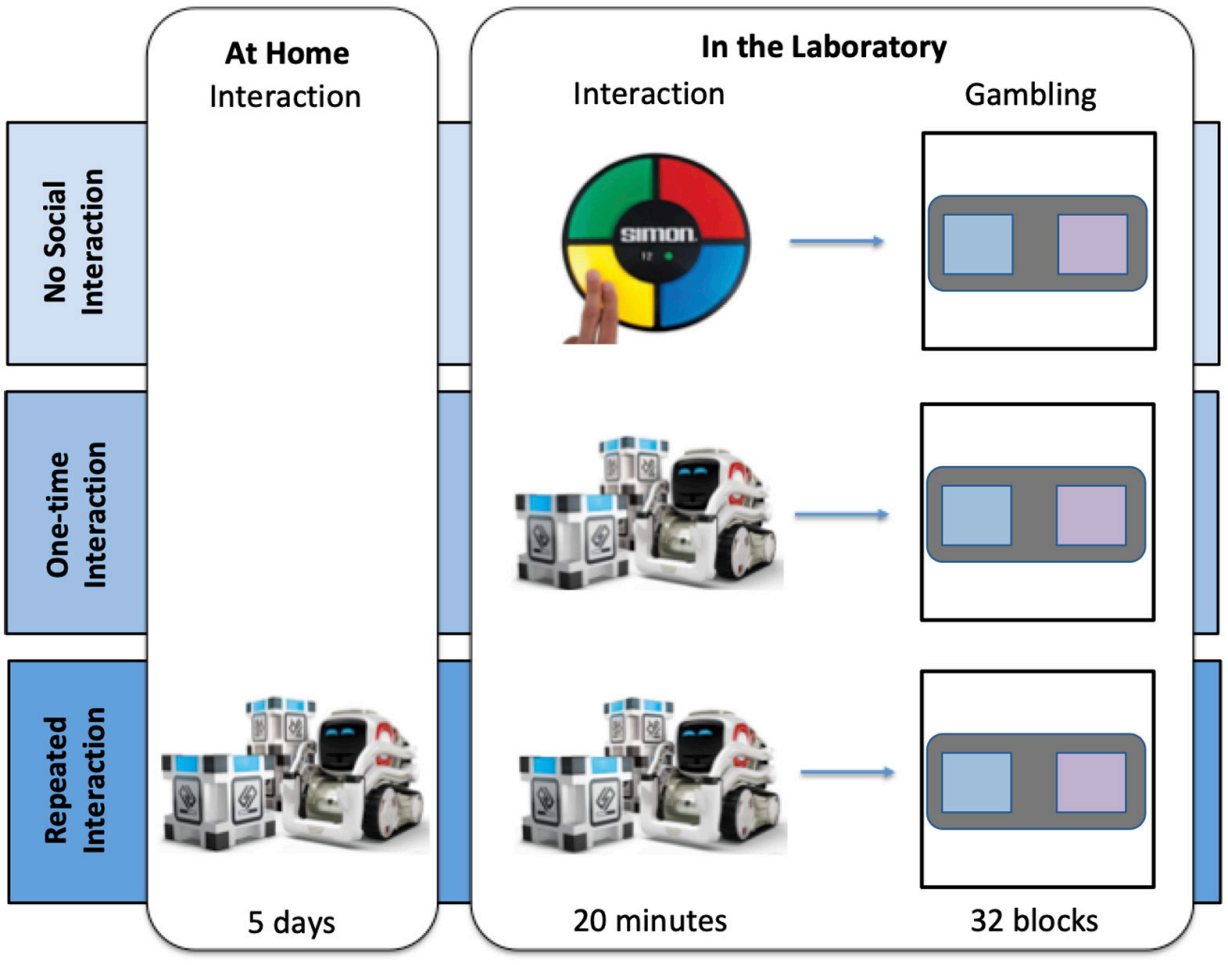
**Overview of the three interaction conditions:** Participants in the no interaction condition arrived to the lab and played the Simon Says game before completing the gambling task. Participants in the one-time interaction arrived to the lab and interacted with Cozmo once before completing the gambling task. Participants in the repeated interaction condition came in at the beginning of the week and were given a Cozmo robot to take home. They were instructed to interact with Cozmo at least 20 minutes per day. No other restrictions were made on their interaction. On the fifth day, they came back to the lab and then completed the gambling task.

Participants were assigned to one of three interaction conditions: (i) ***no interaction*** where participants did not interact with Cozmo prior to the gambling task but played the Simon Says game once for 20 minutes instead; (ii) ***one-time interaction*** where participants interacted once for 20 minutes with Cozmo prior to the gambling task and (iii) ***repeated interaction*** where participants interacted with Cozmo over the course of five days for a minimum of 20 minutes a day in their homes. In the *no social interaction* and *one-time social interaction*, participants performed the interaction task (see below for more details) and immediately afterwards completed the gambling task. In the *repeated social interactions* condition, participants took Cozmo home and interacted with it for at least 20 minutes every day (see below for more details). On day number 5, they came back to the lab and performed the gambling task (after a refresher of the instructions). At the end of the experiment, participants were debriefed and thanked for their participation. A timeline of events for the three interaction conditions can be found in ***Figure 2***.

### Interaction Conditions

In the ***no-interaction*** condition, participants played the *Simon Says* game for about 20 minutes, which required them to imitate a sequence of tones and lights presented on an electronic device; see ***Figure 1***. Every time the participant successfully imitated a sequence, the game would add an extra tone/light to the sequence, which made the game more challenging and gave participants an idea of how well they were performing throughout the game.

In the ***one-time interaction*** condition, participants played two games with Cozmo for about 20 minutes through the Cozmo app: *Keep Away* and *Quick Tap*. On a given trial of *Keep Away*, participants held one of the cubes in front of Cozmo (within the robot’s reaching distance). In the meanwhile, Cozmo attempted to tap the top of the cube: if participants managed to pull the cube away before Cozmo tapped the top, they earned a point; if Cozmo was faster and tapped the cube before it was removed, Cozmo earned a point. In *Quick Tap*, participants played a color matching game with Cozmo involving two cubes: at the beginning of the game, one cube was placed in front of the participant and the other was placed in front of Cozmo (i.e., Cozmo and the participant faced each other with the cubes in between). On a given trial, the cubes would light up with a specific color (either same or different colors). If both cubes showed the same color (e.g., both cubes light up with the color blue), both parties are asked to tap the top of the cube in front of them as fast as possible (i.e., Go-trial). However, if both cubes lit up in red or showed different colors, both parties were asked to refrain from tapping (i.e., Nogo-trial). Participants won the round if (i) they tapped faster than Cozmo on a Go-trial or (ii) correctly withheld their response on a Nogo-trial (only if Cozmo incorrectly taps its cube at the same time). Cozmo won the round if (i) it tapped faster than participants on a Go-trial or if participants do not tap on a Go-trial, (ii) if participants incorrectly tap on a Nogo-trial (only if Cozmo correctly withheld its response at the same time). Neither the participant nor Cozmo received a point if (i) both Cozmo and the participant failed to tap on a Go-trial and/or (ii) both Cozmo and the participant correctly withheld their response on a Nogo-trial. The order in which participants played *Keep Away* and *Quick Tap* was counterbalanced across participants. All of the games were prompted and initiated by the researcher by controlling the app via the tablet.

**Figure 3:**
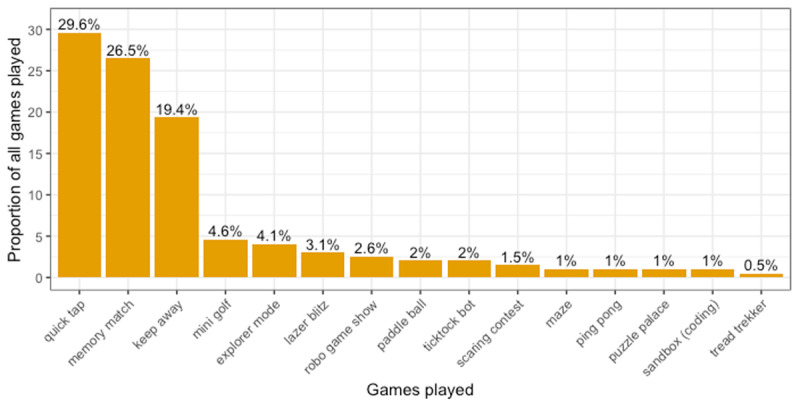
**Proportion of games played:** The graph illustrates participants’ reported proportion of games across all participants. The majority of the games played included “Quick tap”, “Memory match” and “Keep away”.

Participants in the ***repeated interaction*** condition interacted with Cozmo over the course of five days in their homes. They were free to choose (i) how they wanted to interact with Cozmo (all features and games in the app were possible) and (ii) for how long (with a minimum of 20 minutes every day). The five-day period started with participants coming to the laboratory to receive “their” Cozmo and to receive a short tutorial, which was supposed to familiarize them with the robot and the app. After providing consent, the researcher showed them how to use the app and the tablet to prompt Cozmo’s actions and games. After completing the tutorial, participants were asked to explore the app and play with Cozmo for 20 minutes in the lab (to make sure that all participants were able to use the platform on their own). Afterwards, they were instructed about the “at home” part of the study: they were told that they could choose how to engage with Cozmo and for how long each day, but that they should (i) interact with Cozmo for at least 20 minutes every day, and (ii) document their interactions (type and length) via an online questionnaire to ensure that they interacted with Cozmo daily for at least 20 minutes; no other constraints were provided, see ***Table 1*** and ***Figure 3***. for the average duration per day and percentage of games played. Finally, participants were asked to come back to the lab on day 5 to return the robot and complete the gambling part of the study.

**Table 1:** Average reported duration of interaction per day.


DAY	AVERAGE DURATION	SD

1	39.3	14.0

2	35.2	7.5

3	36.8	9.9

4	34.8	7.6

5	34.5	4.9


*Note*: Duration reported is in minutes.

### Gambling Task

After the interaction phase, all participants performed a gambling task that required them to either gamble for themselves (“Self”) or Cozmo (“Cozmo”) by determining which of two differently colored squares (e.g., blue and orange; presented on the horizontal midline of a screen, left and right of a centrally presented fixation cross) produced a winning outcome with a higher frequency. Participants completed 32 blocks (i.e., 16 for Self and 16 for Cozmo) total with a break at the mid-point of the experiment; each block consisted of 20 trials. Whether participants gambled for Self or Cozmo was announced at the beginning of each block by presenting either “Self” or “Cozmo” on the screen; “Self” and “Cozmo” blocks were randomized throughout the experiment. Although the colors of the squares changed after each block (together with the associated likelihood of producing a winning outcome), one color was always associated with a 60% chance of winning and the other color was associated with a 10% chance of winning.

**Figure 4:**
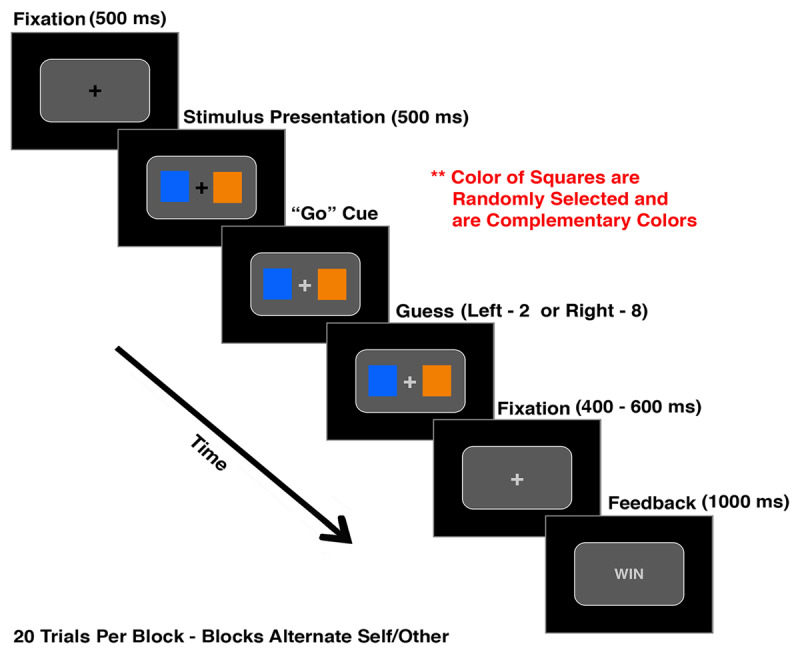
**Trial sequence of the gambling task:** At the beginning of each trial, a black fixation cross was presented centrally for 500 ms, followed by the presentation of two differently colored squares - left and right of the fixation cross. After another 500 ms, the fixation cross changed its color from black to white, which indicated to participants that they could now make their choice by either pressing “2” for the left square or “8” for the right square. After participants made their selection, the fixation cross was presented again for a jittered time interval of 400 to 600 ms. Afterwards, participants received feedback if they lost (“LOSE”) or won (“WIN”) the trial. “Self” versus “Cozmo” was blocked in this experiment and was only shown once at the beginning of a block (not shown).

The trial sequence is shown in ***Figure 4***. Each trial started with the presentation of a central, black fixation cross for 500 ms. The two differently colored squares would then appear left and right of the fixation cross. Although the color scheme would be randomly selected for each block, the colors of the squares would always be complementary colors (e.g., orange and blue); the same two colors were used for all 20 trials within a given block. After the colored squares had been presented for 500 ms, the fixation cross would change its color from black to light grey to indicate to participants that they could now start to gamble (i.e., pick the square they believed was associated with the higher chance of winning). The squares remained on the screen until participants responded with either the “2” key (with their left index finger) to select the square on the left or the “8” key (with their right index finger) to select the square on the right. The probability of each colored square being presented on the left or right side of the screen was equi-probable. After participants submitted their response, feedback would be presented for 1000 ms to inform them about the gambling outcome (i.e., “WIN” or “LOSE”). If participants selected a square prior to the change of the color of the fixation cross, the trial was not included in the analyses. The inter-trial interval (ITI) was jittered between 400–600 ms.

## Analysis

The impact of different interaction modes on feedback processing during the gambling task was analyzed by examining participants’ learning performance over time (i.e., within a block) using a log-log growth curve mixed-model. The growth model examined differences in learning rates as a function of *Condition* (No- vs. One-time vs. Repeated interaction) and *Recipient* (Self vs. Cozmo); both factors were dummy coded. The growth model predicted whether participants chose the color associated with the higher probability of winning or the color associated with the lower probability of winning (i.e., a dichotomous outcome). Interactions between the dummy coded variables and the growth variable examined differences in learning behavior over time between the conditions. To construct the random-effects part of the log-log growth curve, we used a nested model comparison approach to compare four different models to a model that only included the intercept as a predictor, which was the reference of comparison. The models included a log-log growth model that varied the intercept for each participant, a log-log growth model that varied the intercept for each participant and each trial, a log-log growth model that varied the intercept for each participant, each trial and each block and finally, a log-log growth model that varied the intercept for each participant, each trial, each block and a random intercept for the actual score of the gambling task. The nested model comparison allows us to test if the models fit significantly different from the intercept-only model using a Chi-squared test. We then examine the fit indices of the models to tell which of the significantly different models fits the data best. The fixed effects equation can be found in Equation 1 below.

1 ln\left( \gamma \right) = ln\left( {{\beta _0}} \right) + ln\left( {{\beta _1}{X_1}} \right) + {\beta _2}{X_2} + {\beta _3}{X_3} + ln\left( {{\beta _4}{X_1}} \right)\;{X_2} + ln\left( {{\beta _5}{X_1}} \right)\;{X_3} + ln\left( {{\beta _6}{X_1}} \right)\;{X_2}\;{X_3}

## Results

The nested model comparison showed that the model that varied the intercept for each participant (*χ2* (11) = 1204.52, *p* < .001, AIC = 35332, BIC = 35441, –LL = –17653), the model that varied the intercept for each participant and each trial (*χ2* (1) = 4.98, p = .02, AIC = 35329, BIC = 35447, –LL = –17650), the model that varied the intercept for each participant, trial and block (*χ2* (1) = 136.29, *p* < .001, AIC = 35195, BIC = 35321, –LL = –17582) and the model that varied the intercept for the each participant, trial, block and participants’ overall score (*χ2* (1) = 3722.04, *p* < .001, AIC = 31475, BIC = 31609, –LL = –15721) all fit significantly in comparison to the reference model (i.e., the intercept-only model). The fact that the model that varied the intercept for each participant, trial, block and participants’ overall score had the lowest fit indices for all of AIC, BIC and the log likelihood suggests that it is the model that fits the data best. As such, we report the findings from this model.

Results of the growth model are summarized in ***Table 2***; the most important main and interaction effects are highlighted here: (1) *Condition* (One-time vs. Repeated) showed a significant main effect of learning overall. This suggests that participants who were exposed to repeated interactions with Cozmo learned more overall across all blocks and trials by choosing the correct color more often compared than those in the one-time interaction condition: *b* = –.44, *SE* = .16, *z*(33039) = –2.65, *p* < .01. This effect did not reach statistical significance for the One-time vs. No interaction contrast: *b* = –.34, *SE* = .17, *z*(33039) = –1.95, *p* = .051. Although these mean differences are evident between the repeated and the one-time interaction conditions, they do not inform about learning rates.

**Table 2:** Results of the log-log growth curve model.


VARIABLE	B	SE	Z VALUE	*P*

Intercept	–.28	.37	–.75	.45

Growth Curve	.45	.04	10.14	<.001

Condition: *Repeated interaction vs. One-time interaction contrast*	–.44	.16	–2.65	<.01

Condition: *No interaction vs. One-time interaction contrast*	–.34	.17	–1.95	.051

Recipient: *Self vs. Cozmo contrast*	.08	.12	.62	.53

Growth × Condition: *Repeated interaction vs. One-time interaction contrast*	.28	.05	5.06	<.001

Growth × Condition: *No interaction vs. One-time interaction contrast*	.28	.06	4.81	<.001

Growth × Recipient: *Self vs. Cozmo contrast*	–.03	.05	–.58	.56

Condition × Recipient: *Repeated interaction vs. One-time interaction for Self vs. Cozmo contrast*	.23	.17	1.32	.18

Condition × Recipient: *No-interaction vs. One-time interaction for Self vs. Cozmo contrast*	–.08	.19	–.43	.66

Growth × Condition × Recipient: *Repeated interaction vs. One-time interaction for Self vs. Cozmo contrast*	–.17	.07	–2.18	.02

Growth × Condition × Recipient: *No-interaction vs. One-time interaction for Self vs. Cozmo contrast*	.01	.08	.2	.83


*Note*: Significance testing was based on 33039 Degrees of Freedom. The “b” denotes the variable estimate and the “SE” denotes the Standard Error of the estimate.

**Figure 5:**
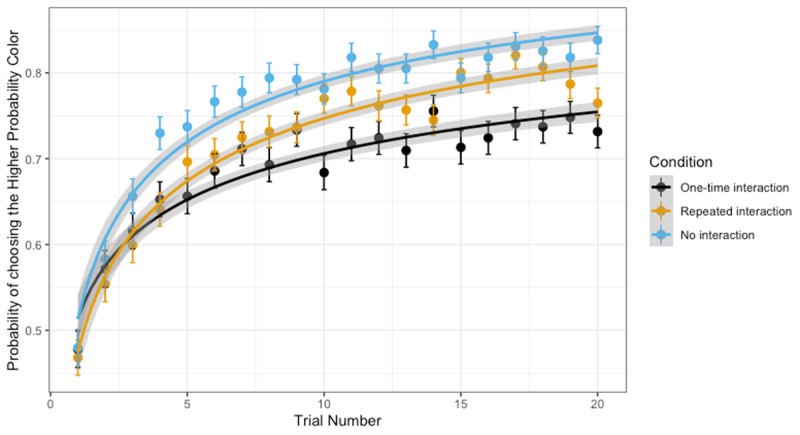
**Learning rates as a function of condition:** Growth curve models allow us to examine differences in learning the higher probability outcome between conditions. The figure illustrates the significant *Growth X Condition* interactions, illustrating that learning rates were significantly different between the No-interaction and the One-time Interaction condition and the One-time interaction vs. the repeated interaction condition. The differences were such that those in the No interaction learned the fastest, followed by those who had repeated interactions and then those who had a one-time interaction with Cozmo. The points in the graph illustrate the percentage of picking the higher probability outcome.

**Figure 6:**
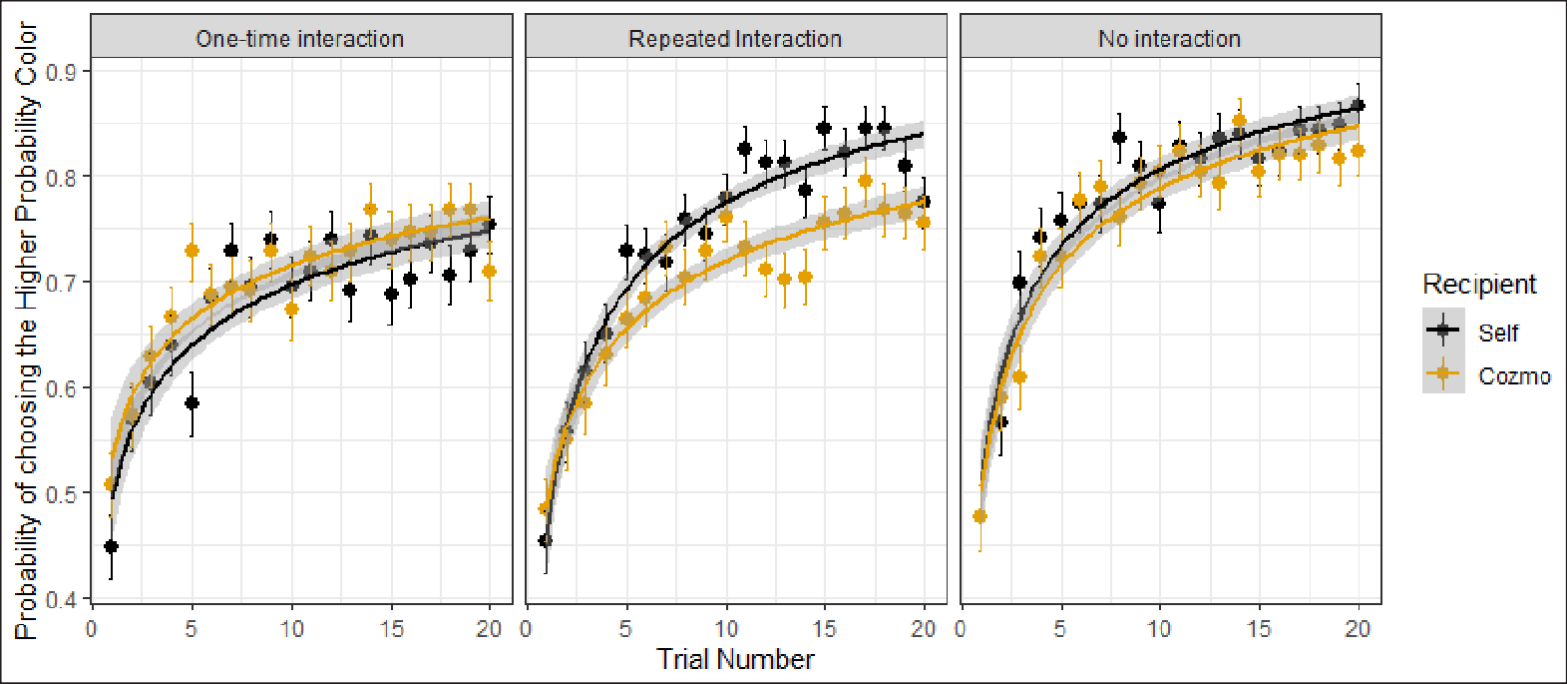
**Learning rates as a function of condition and recipient:** The growth curve model showed significant learning differences in the *Growth* x *Recipient* x *Condition* interaction such learning rates were faster for Self vs. Cozmo in the repeated interaction condition compared to the one-time interaction condition. However, no differences between Self vs. Cozmo were shown between the one-time interaction and the no interaction condition; points represent percentages of picking the higher probability outcome.

(2) *Condition* (No vs. One-time vs. Repeated) had a significant impact on learning rates (i.e., changes in likelihood of choosing the square with the higher chance of winning over the course of one block), as indicated by a significant growth function x *Condition* interaction (for the repeated interaction vs. one-time interaction contrast: *b* = .28, *SE* = .05, *z*(33039) = 5.06, *p* < .001; no interaction vs. one-time interaction contrast: *b* = .28, *SE* = .06, *z*(33039) = 4.81, *p* < .001). Specifically, learning the contingency between color and chance of winning was fastest in the no interaction condition, followed by the repeated interaction condition, followed by the one-time interaction condition (***Figure 5***).

(3) *Recipient* (Self vs. Cozmo) did not significantly impact learning rates across conditions, as indicated by an insignificant growth function x *Recipient* interaction (*b* = –.03, *SE* = .05, *z*(33039) = –.58, *p* = .56). However, learning rates were faster for Self vs. Cozmo in the repeated interaction condition compared to the one-time interaction condition, as indicated by a significant growth function x *Recipient* x *Condition* interaction (for the repeated vs. one-time contrast: *b* = –.17, *SE* = .07, *z*(33039) = –2.18, *p* = .83). There was no Self vs. Cozmo difference in learning rates in the no vs. one-time interaction conditions, as is indicated by a non-significant growth function x *Recipient* x *Condition* interaction (for the no interaction vs. one-time interaction contrast: *b* = .01, *SE* = .08, *z*(33039) = .2, *p* = .83);^3^ see ***Figure 6***.

## Discussion

The goal of the present study was to examine the impact of prior social interactions with a robot on feedback processing embedded in a paradigm with a dynamic (“online”) interaction component. In the social interaction conditions, participants were asked to familiarize themselves with the robot, Cozmo, by either interacting with it for 20 minutes (one-time interaction condition) or for five consecutive days (repeated interaction condition) before performing the gambling task. In the control condition (no social interaction), participants did not familiarize themselves with Cozmo before performing the gambling task but played the game *Simon Says* instead. In all conditions, participants gambled for themselves (i.e., reward goes to participant) or Cozmo (i.e., reward goes to Cozmo).

The findings reveal that the presented hybrid paradigm separating the dynamic (but unstructured) interaction component from the controlled (but structured) measurement component is suitable to detect differences in the effect of different familiarization durations on feedback-based learning without unnecessarily restricting the interaction component to a pre-scripted sequence. The results show that learning contingencies between the square color and chance of winning was fastest in the no interaction condition, followed by the repeated interaction condition, and the one-time interaction condition. Furthermore, a significant difference was found in feedback-based learning between “Self” and “Cozmo” in the repeated interaction condition showing that the paradigm was sensitive to the experimental manipulation of both independent variables (i.e., recipient and condition). This is important to note given that dynamic paradigms (i.e., paradigms, where interaction and measurement take place during a live interaction) often place constraints on the interaction (e.g., large number of repetitions required, interruptions due to measurements being taken) and are difficult to implement (e.g., sending EEG markers in real-time). This is particularly the case when objective behavioral (e.g., reaction times) or neurophysiological (e.g., event-related potentials) measures need to be obtained since recording such measures often requires pre-defined sequences or at least a certain number of critical events (e.g. a human/robot showing a certain behavior), which is often challenging to realize in a fully dynamic interaction and can have negative consequences in terms of comparability and data analysis (e.g., different number of critical events for each participant). The paradigm presented here shows that a “hybrid” solution, that is: the combination of an unscripted interaction component and a scripted measurement component, is worth considering as it does not place significant constraints on the social interaction, but allows behavioral and neurophysiological correlates of social cognition to be obtained with high internal validity. A few early studies pioneering this approach have shown its feasibility for behavioral (current study), neurophysiological (e.g., Abubshait et al., 2021; “hybrid” with an ERP component) and neural (e.g., Cross et al., 2019; “hybrid” with fMRI component) outcome measures.

Two findings require further discussion: (1) learning rates were lower in the one-time interaction than in the repeated interaction condition but highest in the no social interaction condition, and (2) significant differences in feedback-associated learning between “Self” and “Other” were observed only in the repeated interaction condition, but not the one-time or the non-social interaction conditions. These findings suggest potential effects of prior familiarization specific to the robot (i.e., specific effects on the “Self” vs. “Other” condition) might not manifest after a one-time familiarization period, but only when people are more familiarized with robots. While the current results do not allow us to explain all this variance in the data, certain hypotheses (which need to be tested in future experiments) that are based on prior research seem reasonable. For instance, it is possible that the differences in learning between the one-time and repeated social interaction condition is due to a “novelty effect” associated with the robot (Lemaignan et al., 2014) that increases participants’ engagement in the one-time interaction condition but has already washed out after repeated interactions. This interpretation would be in line with previous studies that have suggested that enriched environments modify response to feedback and negatively impact learning due to increased arousal and positive affect (Lohse et al., 2020). It would also be in line with other studies showing that artificial social agents like avatars or robots often cause a distraction in learning environments, which can hinder people’s ability to learn (Kennedy et al., 2015; Momen et al., 2016; Yadollahi et al., 2018). These interpretations are further supported by the fact that feedback-associated learning in the one-time interaction condition was attenuated for “Self” and “Cozmo” instead of being specific to the “Cozmo” condition (as would be expected for a familiarity effect). Future studies will have to be conducted to examine these hypotheses further.

Two challenges for future versions of this paradigm will be to (1) determine which modifications are necessary to replicate the Self-Other difference in feedback processing reported in the original version of the paradigm, and (2) examine the effect of different familiarization protocols (e.g., different durations and/or interaction modes) on feedback processing. Both challenges are related to the fact that the Self-Other difference in feedback processing in the gambling task was not observed in two out of three conditions in the current experiment. Prior work suggests that better responses to feedback for Self vs. Other is explained by an increased intrinsic motivation to win when positive rewards are assigned to oneself versus an unknown player (Hassall et al., 2016; Wilhelm et al., 2019), and been shown to vanish when the “Other” is a known friend versus an unknown stranger (indicating that the “relationship” between the gambler and the recipient of a positive outcome might play a significant role in feedback processing; Leng & Zhou, 2010). Thus, one would have expected a significant Self-Other difference in the control condition (no familiarization), but not in the familiarization conditions.

While the current experiment does not allow any data-driven interpretations, we suspect that the reported findings might be impacted by the participants’ affective state since positive emotions have been shown to impact outcome monitoring (Bakic et al., 2014). Specifically, it is possible that the fact that the participants played engaging games (Simon Says and playing with Cozmo) in the control and one-time interaction (but not the repeated interaction) conditions immediately before performing the gambling task may have elevated their arousal and in turn impacted feedback processing. Additionally, worse learning outcomes specifically for the “Other” condition after repeated interaction would be in line with recent studies showing that repeated exposure to social robots negatively affects social cognitive processes (Abubshait & Wykowska, 2020). We suggest that reduced feedback monitoring for “Other” could be attributable to devaluation of the reward following repeated interactions with the robot. The current study also does not allow us to completely control for differences in reward valuation of winning a gift card for one’s self vs. upgrades for the robot. However, since prior work has failed to find differences in RewP amplitudes for “Self” versus “Other” in the one-time interaction condition (Abubshait et al., 2021), this suggests that the reward values for these two conditions were comparable after a short interaction. This interpretation is also supported by other work that has shown that RewP amplitudes are sensitive to the magnitude of the reward in the gambling task (Hassall et al., 2016). While it is possible that the reward values for “Self” and “Other” did not differ in the one-time interaction condition, it might be that changes in affect over the course of repeated interactions with the robot specifically impacted the degree to which rewards were valued for “Self” vs. “Other”.^4^ Whether participants’ attitudes towards Cozmo were indeed negatively impacted in the repeated interaction condition and whether this potential decrease in positive emotions is strong enough to impact feedback processing warrants further investigation.

We would like to point out that the findings need to be interpreted with care due to a few limitations. First, although we did observe significant effects of all experimental manipulations on the obtained outcome measures, a sample size of 60 participants can be considered small for a three-factor mixed design; this makes it necessary to ensure that the reported findings can be replicated with larger sample sizes. Second, due to the lack of systematic investigations of social cognition in long-term human-robot interactions, it is difficult to empirically determine what the appropriate duration of a familiarization period is supposed to be in order to label an interaction “long-term”. While a duration of five days, as was chosen in the current study, is longer than is reported in most human-robot interaction studies, it is unclear whether this time frame is long enough to induce sustained changes in perceptions of and behaviors towards robots (and to call the interaction “long-term”). Given how important it is to investigate mechanisms of social cognition in human-robot interaction in naturalistic settings over representative periods of time (Dautenhahn, 2007; Giusti & Marti, 2006), it is essential for the field of social robotics to establish systematic standards (e.g., type of tasks and robots, interaction protocols, duration, etc.) of examination (e.g. see Chaudhury et al., 2020; *preprint*; for a tutorial on how to use the robot Cozmo for HRI research).

The reported findings have implications for the fields of social cognition and human-robot interaction. First, the results show that hybrid paradigms that separate the interaction and measurement components are sensitive to examine the impact of social interactions on social-cognitive parameters and might be considered as a pragmatic compromise if fully dynamic paradigms are not feasible. Second, the results indicate that the impact of repeated interactions with an embodied robot on social cognition is multidimensional and changes over time, which requires studies in human-robot interaction to incorporate a repeated measures component. Third, although it is to be expected that social robots will be able to display more sophisticated social behaviors in the future, it seems that current social robot platforms might not be sophisticated enough to engage adult participants positively over longer periods of time. This could lead to negative attitudes towards the robot once the novelty effect has vanished. Despite these challenges, being able to use affordable, easy to program and emotionally engaging robots to examine social cognition in human-robot interaction is an exciting new avenue and will open new possibilities for empirical research in the future.

## Data Availability

Behavioral data are made available and can be found on *osf.io/6dfky/*.

## Footnotes

Cozmo is the ideal platform to examine social-cognitive processes in human-robot interaction due to its affordability, ease of use, socio-emotional expressiveness, availability of source code and accessibility of interaction data (see Chaudhury et al., 2020; *preprint*; for a tutorial), and as such is increasingly used in psychology and computer science research (Charrier et al., 2019; Ciardo et al., 2020; Cross et al., 2019; Hsieh et al., 2020; Lefkeli et al., 2020; McNeill & Kennington, 2019; Pelikan et al., 2020).

Please note that in addition to the behavioral data, we collected EEG data in all three conditions. The EEG data from the no (NI) and the one-time (OTI) interaction condition are reported in Abubshait et al. (2021). The EEG specific procedures are described there. The EEG data of the repeated interaction (RI) condition has not been published yet.

We ran an additional analysis to examine the moderating effect of the reported time of interacting on learning rates for self vs. cozmo for those in the repeated-interaction condition, however, the model did not converge and as such, the estimates are deemed unstable. Therefore, we opted not to report the model.

Please note that although it cannot be ruled out that differences in reward relevance (i.e., being rewarded with the chance of winning a gift card vs. winning upgrades for Cozmo) might play a role in the current paradigm, one would expect this to equally impact all three familiarity conditions and can therefore not explain Self-Other differences in learning in the repeated interaction condition only.
